# New-Onset Psychosis Following COVID-19 Infection

**DOI:** 10.7759/cureus.17904

**Published:** 2021-09-12

**Authors:** Saral Desai, Batool Sheikh, Louis Belzie

**Affiliations:** 1 Department of Psychiatry, Brookdale University Hospital Medical Center, Brooklyn, USA

**Keywords:** neuropsychiatric manifestations, psychiatric symptoms, coronavirus disease, novel coronavirus, psychiatry, remdesivir, systemic steroids, psychosis, covid 19

## Abstract

Previous studies have suggested that some individuals experience neuropsychiatric symptoms following coronavirus disease 2019 (COVID-19) infection. We describe a case of new-onset psychosis following COVID-19 infection in a 55-year-old female with no prior psychiatric history. The patient started exhibiting symptoms of COVID-19 infection three weeks prior and was treated in the hospital with 4 L oxygen, dexamethasone 6 mg, and remdesivir therapy for seven days. Throughout her hospital stay, the patient had no neuropsychiatric symptoms. During her last week of stay, she was solely getting oxygen at home before presenting to the emergency department (ED) with severe psychosis. Her COVID-19 test in ED presentation was negative, and all potential etiologies for psychosis were ruled out. She was effectively treated for two weeks with 10 mg haloperidol and 1000 mg sodium valproate daily, followed by outpatient care. While variables such as a family history of bipolar disorder, psychosocial stressors, and steroid medication may have contributed to the patient's presentation, these circumstances alone did not result in neuropsychiatric symptoms in the past. COVID-19 infection may enhance the likelihood of developing neuropsychiatric problems on its own or amplify the effects of risk factors associated with an increased risk of psychosis. Neuropsychiatric consequences of COVID-19 infection may be under- or over-reported in individuals treated with steroids. Further research is necessary to identify individuals at risk of experiencing neuropsychiatric issues owing to COVID-19 infection and the prognosis.

## Introduction

Virus-borne infections are associated with neuropsychiatric complications. HIV infection, for example, may result in depression, manic episodes, psychosis, and anxiety [1]. Additionally, influenza, varicella, herpes, and hepatitis C are linked to depression and anxiety [2]. Severe acute respiratory syndrome coronavirus 2 (SARS-CoV-2) is a new coronavirus, which is commonly known as coronavirus disease 2019 (COVID-19). Although it is most often linked with respiratory symptoms, it might result in neuropsychiatric complications [3,4]. COVID-19 pandemic and resultant financial stress, social isolation due to lockdown measures, and fear response to the pandemic have led to worse mental health outcomes for susceptible individuals [5]. Although these factors could explain the exacerbation of mental illness or new onset of symptoms, the literature suggests that COVID-19 infection itself can trigger activation of mental illness in susceptible individuals. Previously, a few case reports have described psychosis as a direct consequence of COVID-19 infection [6-11]. We describe a patient with no prior history of mental illness developing severe psychosis after contracting COVID-19.

This article was previously presented as a meeting abstract at the 39th Annual AAPI Convention and Science Assembly on July 2-5, 2021.

## Case presentation

A 55-year-old female with a past medical history of hypertension, type 2 diabetes, and obesity was brought to the emergency department by her family members for concerns regarding her behavior. The patient was discharged from another hospital a week ago, where she was treated for COVID-19 infection using oxygen therapy, steroids, and remdesivir. She received dexamethasone 6 mg IV for seven days. She was discharged with no psychotic symptoms during her hospital stay and continued to receive oxygen therapy at home after steroids and remdesivir were discontinued. The patient became symptomatic with COVID-19 three weeks before her current presentation, and she tested negative on the COVID-19 RNA test performed during the current ED visit. In ED, the patient had a blood pressure of 147/119 mmHg and tachycardia (130 bpm). Her SpO2 saturation was 97%. The patient was without any fever at the presentation. Her lactate dehydrogenase levels were 825 units/L, and her white blood cell count was 4500 cells/mm^3^. She had non-command type auditory hallucinations and delusions of religious nature. She reported hearing God’s voice asking her to save the earth. COVID-19 was also the central theme of such hallucinations/delusions. She was agitated, screaming at staff members, and removing IV lines. She received diphenhydramine 25 mg, haloperidol 5 mg, and lorazepam 2 mg for acute management of symptoms. She was also started on metoprolol 25 mg for management of her hypertension. Infectious disease workup was negative. MRI and CT scans of the brain were unremarkable. The urine drug screen was negative for substances. All etiologies of such presentation were ruled out.

On a follow-up visit the next day, the patient continued to have grandiose delusions, her speech was pressured. She was preoccupied with God, COVID-19, and the Devil. She reported seeing God and reported that God is talking with her during the interview. It was decided to admit the patient to the inpatient psychiatric ward. She was started on haloperidol 10 mg and sodium valproate 1000 mg for treatment. She continued to show improvement in her symptoms. Considering the better side effect profile of second-generation antipsychotics regarding extrapyramidal side effects, she was discharged from the hospital on aripiprazole 10 mg and sodium valproate 1000 mg daily after two weeks of inpatient stay to follow-up at the outpatient clinic.

During her stay, we collected collateral information from multiple sources. The patient was never diagnosed with any psychiatric illness. She never visited a psychiatrist in her lifetime. Family history is positive for bipolar disorder type I in a distant cousin. Her family also has a history of developing psychiatric symptoms following steroid use. However, there is no history of her developing psychiatric symptoms following treatment with steroids in the past. The family members reported that they have never seen abnormal behavior in her before this instance.

## Discussion

Although COVID-19 was previously thought to be a respiratory illness, new research indicates a multi-systemic vascular disease affecting several organs [12]. This cascade of cytokine-mediated inflammation and microvascular injury may cause the post-COVID-19 syndrome, a condition in which patients have a chronic subjective cognitive impairment, sleep problems, and mood symptoms [13]. Virus-induced neuropsychiatric symptoms are not a novel occurrence. For instance, numerous influenza virus strains have been implicated with the development of neuropsychiatric symptoms such as schizophrenia, depression, encephalitis, anxiety, and acute psychosis [14,15]. SARS illness, caused by a different strain of coronavirus, has previously been demonstrated to elicit neuropsychiatric symptoms in individuals [14,15].

Steroid treatment for COVID-19 pneumonia could potentially explain the patient’s new-onset psychosis. However, in our case, on ED presentation, the patient’s lab work did not show leukocytosis that would be expected for individuals currently using corticosteroids. She had high blood pressure, which could be attributable to steroid use, but collateral history confirmed that the patient was poorly compliant with her blood pressure medications. It is also unusual for a patient to not have psychiatric symptoms while receiving steroids for COVID-19 pneumonia and then present with psychosis seven days after cessation of steroid therapy. Although there is a family history of adverse psychiatric effects from steroid use in the patient’s brother, the patient herself did not have such a history of any acute or delayed onset psychiatric symptoms from past steroid use. According to research on SARS patients, the severity of the disease, the dose of steroids, and psychosocial variables led to an increased chance of developing mental symptoms [16]. Thus, one could argue that SARS-CoV-2, the novel coronavirus responsible for the COVID-19 pandemic, would have similar findings. In that instance, our patient's severe psychosis might have resulted from a combination of COVID-19 infection, steroid therapy, and psychological stresses.

A large retrospective cohort study using electronic health record data found that patients with no prior psychiatric history diagnosed with COVID-19 had a greater likelihood of receiving their first psychiatric diagnosis after 14-90 days [17]. Thus, neuropsychiatric symptoms may manifest during both the active phase of COVID-19 infection and the post-infection period. In this case, the patient went to the emergency department with psychosis three weeks following the onset of COVID-19 symptoms. Another intriguing feature of our case is that the patient's psychotic symptoms were unresponsive to antipsychotics alone. A mix of antipsychotics and mood stabilizers helped her symptoms dramatically. She may have been prone to develop neuropsychiatric symptoms due to COVID-19 infection and a family history of bipolar disorder.

## Conclusions

Although COVID-19 is commonly linked with respiratory symptoms, some vulnerable individuals might develop new-onset neuropsychiatric symptoms without a prior history of psychiatric illness. This presentation may be acute throughout the course of the illness or may occur post-infection. While a family history of mental disease, steroid usage for COVID-19 pneumonia therapy, and psychological stresses associated with the COVID-19 pandemic may have had a role in our case. The patient acquired neuropsychiatric symptoms only after acquiring COVID-19 infection. It's possible that COVID-19 had a unique function, either by itself or by enhancing the impact of other neuropsychiatric symptoms-related variables. Our case also highlights the interplay between corticosteroids, COVID-19, and psychosis. Steroid use for the treatment of COVID-19 pneumonia could lead to under- or over-reporting COVID-19-related neuropsychiatric symptoms.

Additional research must identify vulnerable individuals at an elevated risk of acquiring neuropsychiatric problems due to COVID-19 infection and the prognosis of such presentations.
